# Cooperation/supervision of a habit by a cognitive strategy in a goal-directed navigational paradigm

**DOI:** 10.1186/1471-2202-16-S1-P200

**Published:** 2015-12-18

**Authors:** Souheïl Hanoune, Jean-Paul Banquet, Philippe Gaussier, Mathias Quoy

**Affiliations:** 1 EIS Lab, University of Cergy-Pontoise, ENSEA - CNRS, Paris, France

## 

The Stimulus-Response (S-R) theory and Tolman's Cognitive Theory of behavior control both issued from behaviorism in the early 20th century still provide a relevant general framework to account for animal reward-based adaptive behavior. In this paper, we propose a new paradigm for representing and implementing both the cognitive strategy and the S-R habit strategy within a unitary coding frame. Based on a parallel learning of both strategies, the model explains how the fast learning cognitive strategy can supervise and accelerate the slow learning S-R habit strategy; and also how. In late learning stages, the habit strategy can overcome the cognitive. This parallel representation is inspired by the cortico-basal functional loops [1] and the cooperation between the cognitive associative loop, including the dorso-medial striatum and the mPF; and the sensory-motor loop, associated to the sensory motor cortex in relation with the dorso-lateral striatum.

The implementation of S-R habit strategy is based on a neural modified version of the classical Q-learning and is based on the model of [2], emulating the functioning of the sensory-motor loop. The states of the model are represented by hippocampal transitions, representing associations between two consecutive place-cells during the exploration of the environment, learned in the CA1-CA3 regions of the hippocampus. The cognitive strategy is based on a map representation of the environment namely the cognitive map [3]. Based on the association between learned transitions, the cognitive map allows the back-propagation of a reward within a tree, allowing the selection of the shortest path to the goal. While the cognitive map is quickly learned, the Q-values associated with the Q-learning are slower to acquire. On the other hand, the Q-learning tends to be more accurate than the cognitive map when fully learned.

**Figure 1:**
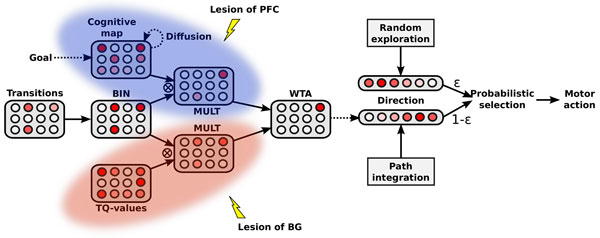
**Cooperative architecture between the cognitive and the S-R habit with a representation of the respective lesions**.

**Figure 2:**
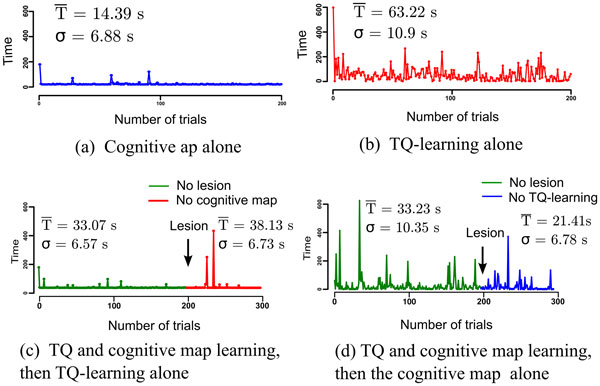
**Statistical results for the sessions of the different groups representing the different situations where the S-R habits trategy is alone, the cognitive strategy is alone, or when one of the two is lesioned after cooperative learning**.

The model exploits this speed difference in its parallel learning. The fast acquisition of the cognitive map allows the robot to quickly choose correct paths to the goal, and thus the time convergence of the Q-learning algorithm is optimized. The cooperation is based on the biasing of the selected transition by the cognitive map and the Q-learning in parallel (see Figure 1). In its early learning stage, the Q-learning biasing is too weak, and the cognitive map is dominant (Figure 2. VS Figure 2b), inducing the supervision of the S-R habit by the cognitive strategy. In the later learning stages, the Q-learning is stronger and more precise. Cooperation of the cognitive strategy and S-R habit enables a faster S-R learning; as shown in Figure 2. The lesion studies (Figure 2c, Figure 2d) show that the system maintains a coherent behavior event after the lesion of either of the structures supporting the two strategies. Also, the time responses highlight the superiority of the habit strategy after over-training (Figure 2.c VS Figure 2.d).
